# Genetic Polymorphisms and Pharmacotherapy for Prostate Cancer

**DOI:** 10.31662/jmaj.2021-0004

**Published:** 2021-03-26

**Authors:** Masaki Shiota, Shusuke Akamatsu, Shintaro Narita, Naoki Terada, Naohiro Fujimoto, Masatoshi Eto

**Affiliations:** 1 Department of Urology, Graduate School of Medical Sciences, Kyushu University, Fukuoka, Japan; 2 Department of Urology, Graduate School of Medicine, Kyoto University, Kyoto, Japan; 3 Department of Urology, Graduate School of Medicine, Akita University, Akita, Japan; 4 Department of Urology, Faculty of Medicine, University of Miyazaki, Miyazaki, Japan; 5 Department of Urology, School of Medicine, University of Occupational and Environmental Health, Kitakyushu, Japan

**Keywords:** androgen metabolism, androgen receptor, genetic polymorphism, pharmacotherapy, prostate cancer

## Abstract

The therapeutic landscape of pharmacotherapy for prostate cancer has dramatically evolved, and multiple therapeutic options have become available for prostate cancer patients. Therefore, useful biomarkers to identify suitable candidates for treatment are required to maximize the efficacy of pharmacotherapy. Genetic polymorphisms such as single-nucleotide polymorphisms (SNPs) and tandem repeats have been shown to influence the therapeutic effects of pharmacotherapy for prostate cancer patients. For example, genetic polymorphisms in the genes involved in androgen receptor signaling are reported to be associated with the therapeutic outcome of androgen-deprivation therapy as well as androgen receptor-pathway inhibitors. In addition, SNPs in genes involved in drug metabolism and efflux pumps are associated with therapeutic effects of taxane chemotherapy. Thus, genetic polymorphisms such as SNPs are promising biomarkers to realize personalized medicine. Here, we overview the current findings on the influence of genetic polymorphisms on the outcome of pharmacotherapy for prostate cancer and discuss current issues as well as future visions in this field.

## Introduction

Androgen-deprivation therapy (ADT) with or without first-generation anti-androgen agents has been the gold standard as primary pharmacotherapy for treatment-naïve prostate cancer ^(1)^. Recently, the therapeutic landscape of pharmacotherapy for prostate cancer patients has been greatly evolving. Second-generation anti-androgen agents such as enzalutamide, apalutamide, and darolutamide as well as the CYP17 inhibitor abiraterone have been developed for castration-resistant prostate cancer (CRPC) ^(2)^. Although these drugs were initially developed for the treatment of CRPC, enzalutamide, apalutamide, and abiraterone have expanded for use in hormone-sensitive prostate cancer (HSPC) ^(3)^. In addition, taxane chemotherapy (docetaxel and cabazitaxel) and radioisotopes (radium-223) have been applied for the treatment of CRPC, and docetaxel has been indicated for HSPC ^(2)^. Thus, multiple therapeutic options for CRPC and HSPC are available. Therefore, useful biomarkers to identify patients that are suitable candidates for these treatments are required to maximize the efficacy of pharmacotherapy.

**Figure 1.**
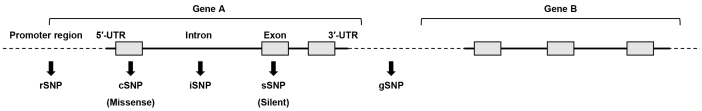
Schematic of single-nucleotide polymorphism (SNP) types according to the location and function. UTR, untranslated region.

Genetic polymorphisms are considered one of the most promising biomarkers for the realization of personalized medicine ^(4)^. Genetic polymorphisms are inter-individual differences in germline DNA and defined as differences in genomic sequences between individuals that occur at a frequency of 1% or more in a population. Most genetic polymorphisms are single-nucleotide polymorphisms (SNPs), and polymorphisms are also detected in repeated sequences such as microsatellites. SNPs are observed at a frequency of ~1 in 1000 nucleotides, and more than 2 million SNPs exist in the entire human genome. SNPs are classified into the following types according to their function: regulatory SNPs (rSNPs), which are located in promoter regions; coding SNPs (cSNPs), which are located in exons and cause an amino acid substitution; silent SNPs (sSNPs), which are located in an exon but do not cause an amino acid substitution; intron SNPs (iSNPs), which are located in introns; and genome SNPs (gSNPs), which are located in intergenic regions (Figure 1). Accordingly, rSNPs and cSNPs are likely to change gene expression and protein function, which results in functional and phenotypic differences, respectively. In addition, sSNPs and iSNPs may affect expression levels of genes. Conversely, gSNPs are speculated to not play a direct functional role, but these may serve as genomic markers linked with distinct functional SNPs.

Genetic polymorphisms can cause various phenotypic differences through changes of expression and/or activity in the corresponding gene. Genetic polymorphisms are also associated not only with disease susceptibility but also with treatment outcomes. For example, a genetic polymorphism in *UGT1A1* (UGT1A1*28 and UGT1A1*6), which encodes UDP-glucuronosyltransferase, decreases enzyme activity, and delays the metabolism of SN-38, the active metabolite of irinotecan, which results in a higher incidence of adverse events by irinotecan ^(5)^. A test for genetic polymorphisms in *UGT1A1* has been approved in Japan for patients who will be treated with irinotecan chemotherapy. A SNP in *Nudix hydrolase 15* (*NUDT15*), which encodes the enzyme involved in the metabolism of thiopurines, was shown to be useful in predicting adverse events of thiopurines. A test for genetic polymorphisms in *NUDT15* was recently approved in Japan for patients who will be treated with thiopurines ^(6)^. Thus, the significance of testing genetic polymorphisms including SNPs in medical care has been growing.

Several genome-wide associated studies (GWASs) on prostate cancer susceptibility in large cohorts have been reported, showing the value of hundreds of SNPs with prostate cancer incidence ^(7), (8)^. In addition, various studies have reported the significance of SNPs in the outcome of pharmacotherapy for prostate cancer ^(9)^. An association of genetic background such as race and family history with the outcome of prostate cancer has been shown, which suggests that genetic factors play an important role in pharmacotherapy ^(10), (11)^. In this review, we provide an overview of the current findings on the influence of genetic polymorphisms in pharmacotherapy for prostate cancer and discuss current issues and future directions in this field.

## Genetic Polymorphisms and Primary ADT for HSPC

Aberrant activation of androgen receptor (AR) signaling is one of the main causes by which prostate cancer acquires castration resistance. Therefore, polymorphisms in genes related to the AR pathway may affect the therapeutic efficacy of primary ADT through influencing AR signaling activity ^(9)^. To date, 63 SNPs in 49 genes have been reported to be associated with the outcome of primary ADT for HSPC (Table 1).

**Table 1.** Genetic Polymorphisms Associated with Treatment Outcomes for Hormone-Sensitive Prostate Cancer.

Gene name	Function	rs number	Polymorphism types	Treatment	Validation	Reference
*CYP17A1*	Androgen metabolism	rs6162	sSNP	ADT		^(12)^
		rs743572	rSNP	ADT	Validated	^(13), (14)^
*CYP19A1*	Androgen metabolism	rs1870050	iSNP	ADT	Almost validated	^(12), (15), (16)^
		rs4775936	iSNP	ADT		^(17)^
*HSD3B1*		rs1047303	cSNP	ADT	Validated	^(18)-(22)^
				ADT+Docetaxel		^(22)^
		rs1856888	gSNP	ADT	Almost validated	^(15), (23)^
*HSD17B2*	Androgen metabolism	rs4243229, rs7201637	iSNP	ADT		^(12)^
*HSD17B3*	Androgen metabolism	rs2257157	iSNP	ADT		^(12)^
*HSD17B4*	Androgen metabolism	rs7737181	iSNP	ADT		^(15)^
*AKR1C3*	Androgen metabolism	rs12529	cSNP	ADT	Controversial	^(24), (25)^
*SRD5A2*	Androgen metabolism	rs523349	cSNP	ADT		^(26)^
*SLCO1B3*	Androgen transporter	rs4149117	cSNP	ADT	Validated	^(27)-(29)^
*SLCO2B1*	Androgen transporter	rs1077858	iSNP	ADT	Validated	^(30), (31)^
		rs1789693	iSNP	ADT		^(30)^
		rs12422149	cSNP	ADT	Almost validated	^(29)-(32)^
*GNRH2*	Androgen synthesis	rs6051545	cSNP	ADT		^(33)^
*SHBG*	Androgen-binding protein	rs6259	cSNP	ADT	Controversial	^(34), (35)^
*AR*	Steroid receptor	CAG repeat	Coding region	ADT	Almost validated	^(24), (36), (37)^
*ESR1*	Steroid receptor	rs1062577	rSNP	ADT		^(12)^
*NR3C2*	Steroid receptor	rs5522	cSNP	ADT		^(38)^
*YB-1*	Transcription factor	rs12030724	iSNP	ADT	Validated	^(39), (40)^
*HIF1A*	Transcription factor	rs11549465	cSNP	ADT		^(41)^
*ARRDC3*	Target gene of AR	rs2939244	rSNP	ADT		^(42)^
*FLT1*	Target gene of AR	rs9508016	rSNP	ADT		^(42)^
*SKAP1*	Target gene of AR	rs6054145	rSNP	ADT		^(42)^
*FBXO32*	Target gene of AR	rs7830622	rSNP	ADT		^(42)^
*BNC2*	Target gene of ER	rs16934641	rSNP	ADT		^(43)^
*TACC2*	Target gene of ER	rs3763763	rSNP	ADT		^(43)^
*ALPK1*	Target gene of ER	rs2051778	rSNP	ADT		^(43)^
*LSAMP*	Target gene of NFκB	rs13088089	rSNP	ADT		^(44)^
*CCL17*	Target gene of NFκB	rs223899	rSNP	ADT		^(44)^
*PSMD7*	Target gene of NFκB	rs2387084	rSNP	ADT		^(44)^
*MON1B*	Target gene of NFκB	rs284924	rSNP	ADT		^(44)^
*GSTM3*	Antioxidant	rs7483	cSNP	ADT	Validated	^(45)^
*CAT*	Antioxidant	rs564250	gSNP	ADT		^(45)^
*SLC28A3*	Nucleoside transporter	rs56350726	cSNP	ADT		^(46)^
*LRP2*	Sterol and steroid transporter	rs6433107, rs3944004, rs830994, rs3770613, rs831003	iSNP	ADT		^(47)^
*EGF*	Growth factor	rs4444903	rSNP	ADT		^(48)^
*IRS2*	Growth factor	rs7986346	gSNP	ADT		^(49)^
*TGFBR2*	TGF-β signaling	rs3087465	iSNP	ADT		^(50)^
*BMP5*	TGF-β signaling	rs317027	gSNP	ADT		^(49)^
*IL18*	Cytokine	rs187238	rSNP	ADT		^(51)^
*APC*	Wnt signaling	rs2707765, rs497844	iSNP	ADT		^(52)^
*BGLAP*	Bone metabolism	rs1800247	rSNP	ADT		^(53)^
*EDN1*	Vasoconstrictor	rs1800541, rs2070699	iSNP	ADT		^(54)^
*CASP3*	Apoptosis	rs4862396	gSNP	ADT		^(49)^
*TRMT11*	Methyltransferase	rs1268121, rs6900796	iSNP	ADT		^(55)^
*COMT*	Methyltransferase	rs4680	cSNP	Estramustine phosphate		^(56)^
*KIF3C*	miRNA target site	rs6728684	rSNP	ADT		^(57)^
*CDON*	miRNA target site	rs3737336	rSNP	ADT		^(57)^
*IFI30*	miRNA target site	rs1045747	rSNP	ADT		^(57)^
*PALLD*	miRNA target site	rs1071738	rSNP	ADT		^(57)^
*GABRA1*	miRNA target site	rs998754	rSNP	ADT		^(57)^
*SYT9*	miRNA target site	rs4351800	rSNP	ADT		^(57)^
-	-	rs16901979, rs7931342	gSNP	ADT		^(58)^

ADT, androgen deprivation therapy; AR, androgen receptor; ER, estrogen receptor; NFκB, nuclear factor-κ B; SNP, single-nucleotide polymorphism; TGF, tumor growth factor

**Figure 2.**
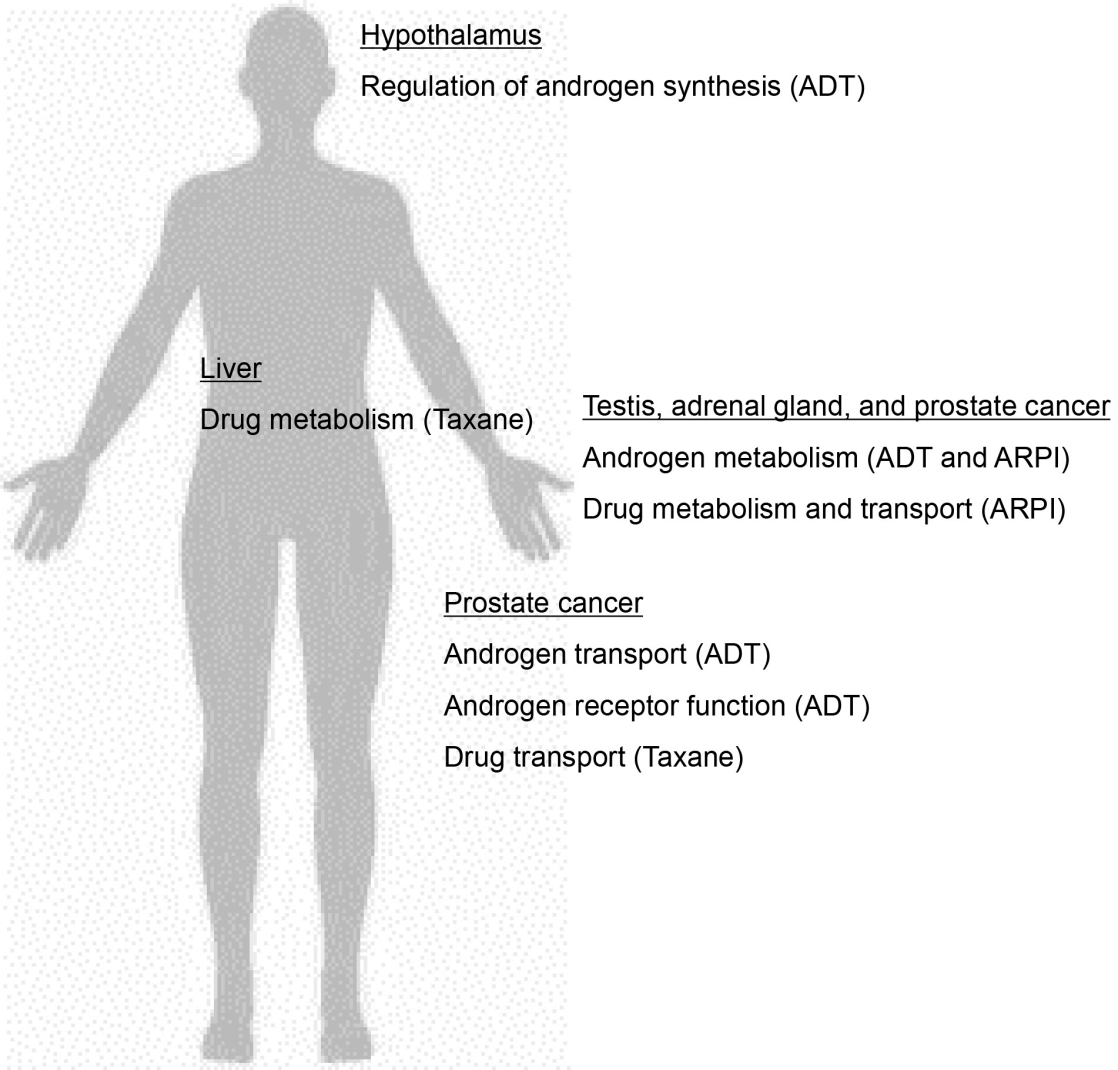
Gene function of single-nucleotide polymorphisms (SNPs) associated with therapeutic effects and adverse events of drug therapy. Underlined organs and treatments in parentheses mean target organ and treatment in which the gene function of SNPs is involved, respectively. ADT, androgen-deprivation therapy; ARPI, androgen receptor-pathway inhibitor.

*De novo* androgen synthesis in prostate cancer cells is a major source of androgen under castrated condition during ADT and is shown to play an important role in the progression to CRPC ^(59)^. Multiple studies have indicated the association of SNPs in genes involved in androgen metabolism, including *CYP17A1*, *CYP19A1*, *HSD3B1*, *HSD17B2*, *HSD17B3*, *HSD17B4*, *AKR1C3*, and *SRD5A2*, with the outcome of ADT (Figure 2). For example, a cSNP (rs1047303, 1245A>C, N367T) in *HSD3B1*, which encodes 3β-hydroxysteroid dehydrogenase 1 (3β-HSD1), results in a variant of 3β-HSD1 with high activity, and the prognosis of carriers of this variant is poor ^(18), (19), (20), (21), (22), (60)^. The prognostic impact of the cSNP (rs1047303) in* HSD3B1* in the United States was validated in an Asian cohort ^(21)^, although variant carriers were rare in Asian patients (~15%) compared with Caucasian patients (~50%) (Table 2). The prognostic impact of the cSNP (rs1047303) in *HSD3B1* was validated in primary ADT plus docetaxel for HSPC ^(22)^. In addition, the prognostic difference by another SNP (rs1856888) in *HSD3B1* was also indicated. Ross et al. initially reported that the variant G allele in rs1856888 was associated with a low risk of disease progression among men in the United States ^(15)^; however, a recent study from the United States showed poor prognosis in patients carrying the variant G allele in rs1856888 ^(23)^. Because of the strong linkage disequilibrium between the SNPs (rs1047303 and rs1856888) in *HSD3B1* ^(23)^, the variant allele in the SNPs (rs1047303 and rs1856888) in *HSD3B1* is likely to be associated with poor prognosis in patients treated with primary ADT. In a study on an iSNP (rs1870050) in *CYP19A1*, Ross et al. reported that the variant C allele in rs1870050 was associated with a high risk of disease progression among men in the United States ^(15)^. However, two recent studies showed a low risk of progression and better prognosis among Asian men with the variant C allele in rs1870050 ^(12), (16)^. In addition, the prognostic significance of an rSNP (rs743572) in the 5′ untranslated region of *CYP17A1* has been shown ^(13), (14)^.

**Table 2.** Outcome and Frequencies of the rs1047303 Variant Allele of HSD3B1.

Outcome	Variant carrier	Number	Frequency carrying a variant allele	Reference
Prostate cancer susceptibility	High	626	48% (AC/CC, US)	^(62)^
Hereditary prostate cancer susceptibility	High	98	53% (AC/CC, US)	^(63)^
Prognosis in primary ADT	Poor	118/137/118	51% (AC/CC, US)	^(18)^
Prognosis in primary ADT	Poor	102	53% (AC/CC, US)	^(19)^
Prognosis in primary ADT	Poor	218	54% (AC/CC, US)	^(20)^
Prognosis in Abiraterone	Insignificant	76	45% (AC/CC, US)	^(64)^
Progression in primary ADT or ADT+Docetaxel	Poor in low volume	475	53% (AC/CC, US)	^(22)^
Prognosis in Ezalutamide or Abiraterone	Poor	266	8% (CC, US/UK)	^(65)^
Prognosis in Ezalutamide or Abiraterone	Poor	547	15% (CC, Canada/Europe)	^(66)^
Prognosis in primary ADT	Insignificant	103	18% (AC/CC, China)	^(67)^
Prognosis in primary ADT	Poor	104	9% (AC/CC, Japan)	^(21)^
Prognosis in Abiraterone	Favorable	99	14% (AC/CC, Japan)	^(21)^

ADT, androgen deprivation therapy; UK, United Kingdom; US, United States

**Figure 3.**
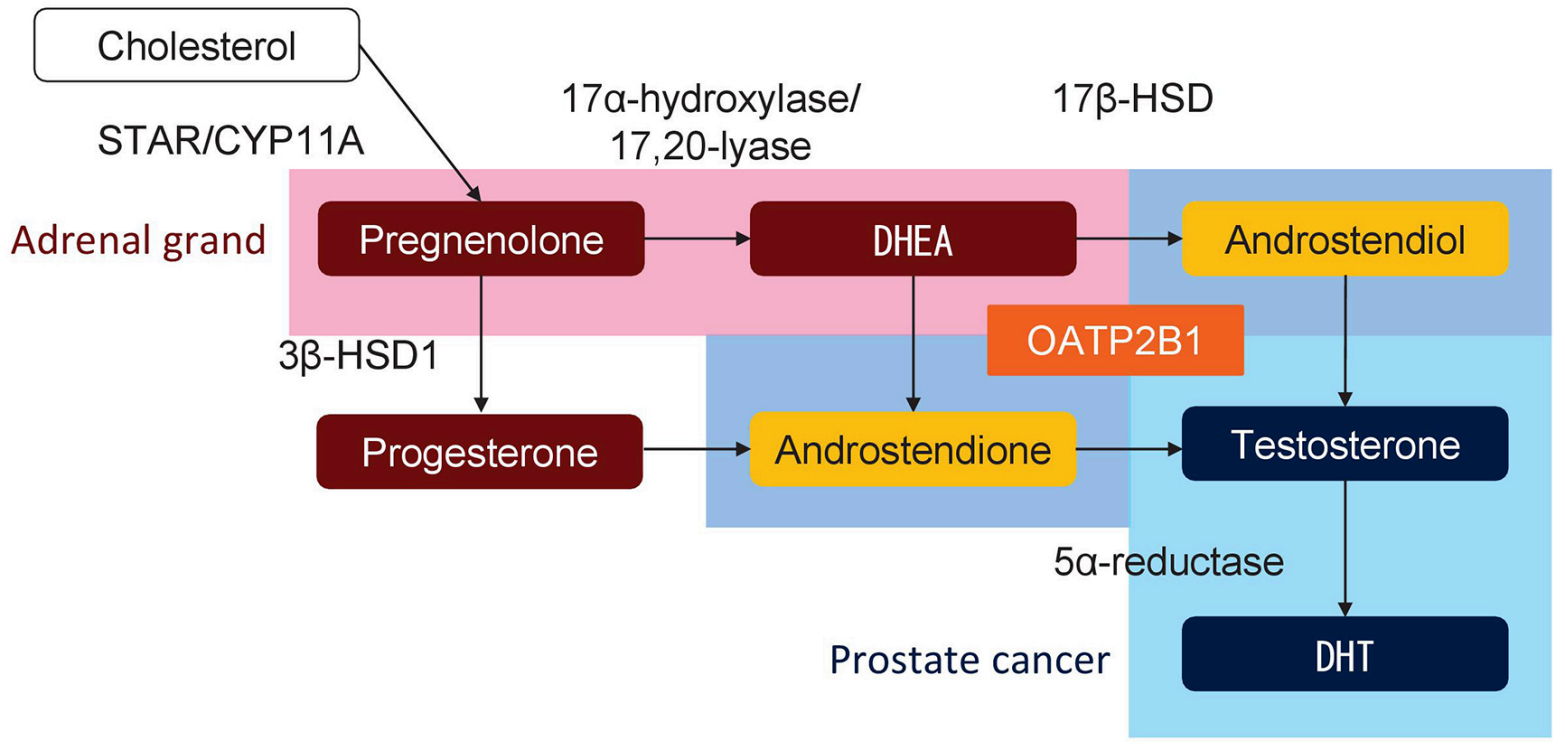
Schematic of molecules involved in androgen synthesis and uptake. The metabolisms surrounded by red, light blue, and blue are mainly processed in adrenal glands, prostate cancer, and both, respectively. OATP2B1 uptakes DHEA into prostate cancer cells. DHEA, dehydroepiandrosterone; DHT, dihydrotestosterone.

In addition to enzymes for androgen metabolism, the pump for androgens such as dehydroepiandrosterone (DHEA) and testosterone also plays a key role in the development of CRPC ^(29)^. SNPs in *SLCO1B3* and *SLCO2B1* genes, which encode proteins responsible for the import of testosterone and DHEA, respectively, were reported to be associated with the prognosis of patients treated with primary ADT ^(27), (28), (29), (30), (31), (32)^. Higher testosterone uptake in patients with the variant allele of the cSNP (rs4149117, 334G>T, A112S) in *SLCO1B3* was shown, and a causal variant of *SLCO1B3* was reported to be associated with poor prognosis ^(27), (28), (29)^. Several studies demonstrated that the cSNP (rs12422149, 935G>A, R312Q) in *SLCO2B1* resulted in higher activity of DHEA-sulfate uptake and the wild-type allele in *SLCO2B1* (rs12422149) was associated with early recurrence and poor prognosis after primary ADT ^(29), (30), (31), (32)^. Another SNP (rs1077858) in *SLCO2B1* was also associated with prognosis ^(30), (31)^. Thus, SNPs in the genes involved in androgen metabolism and uptake in prostate cancer cells play a key role in the progression of prostate cancer through persistent androgen synthesis in prostate cancer under castrated condition (Figure 3).

SNPs in other molecules related to the AR pathway were also shown to have prognostic impact after primary ADT. For example, the CAG repeat in *AR* correlated with prognosis, although null results were also reported ^(24), (36), (37), (61)^. In addition, the iSNP (rs12030724) in *YB-1* that regulates YB-1 expression, which results in AR and AR variant expression, was also associated with the prognosis of Japanese men with advanced prostate cancer treated with primary ADT ^(39), (40)^. The cSNP (rs7483, I224V) in *GSTM3*, which encodes an antioxidant enzyme, was also reported to be prognostic in Japanese patients with nonmetastatic and advanced prostate cancer treated with primary ADT ^(45)^.

## Genetic Polymorphisms and Treatment with Novel AR-pathway Inhibitors (ARPIs) for CRPC

Novel ARPIs such as enzalutamide, apalutamide darolutamide, and abiraterone have been demonstrate to improve survival in patients with CRPC ^(2)^. Because abiraterone is taken up into cells by OATP2B1, which is encoded by *SLCO2B1*, and then metabolized by 3β-HSD and 5α-reductase, the therapeutic effect of abiraterone treatment may depend on the activities of the molecules involved in androgen metabolism and uptake (Figure 2) ^(73), (78)^. Recent reports showed that SNPs in genes involved in androgen metabolism and transport such as *CYP17A1*, *HSD3B1*, *SRD5A2*, and *SLCO2B1* correlate with the outcome of abiraterone treatment (Table 3). An rSNP (rs2486758, -362T>C) in *CYP17A1* was associated with prognosis after abiraterone treatment ^(68), (69)^. In addition, variant carriers of the cSNP (rs1047303) in *HSD3B1* showed poor prognosis after treatment with ARPI ^(65), (66)^. The prognostic impact of the cSNP (rs1047303) in *HSD3B1* for both primary ADT for HSPC as well as ARPIs for CRPC may be because of hyperactive androgen synthesis in variant carriers. The variant allele in *HSD3B1* is expected to lead to increased conversion from abiraterone to the more potent delta-4-abiraterone ^(78)^. Accordingly, the cSNP (rs1047303) in *HSD3B1* was shown to be associated with comparable or better treatment efficacy of abiraterone ^(21), (64)^.

**Table 3.** Genetic Polymorphisms Associated with the Prognosis of Patients with Castration-Resistant Prostate Cancer Treated with Androgen Receptor-Pathway Inhibitors.

Gene name	Function	rs number	Polymorphism types	Treatment	Validation	Reference
*CYP17A1*	Androgen metabolism	rs2486758	rSNP	Abiraterone	Validated	^(68), (69)^
		rs10883783	iSNP	Abiraterone		^(70)^
*HSD3B1*	Androgen metabolism	rs1047303	cSNP	Abiraterone		^(21)^
				Abiraterone or Enzalutamide	Validated	^(65), (66)^
*SRD5A2*	Androgen metabolism	rs523349	cSNP	Abiraterone		^(71)^
*SLCO2B1*	Androgen transporter	rs1077858, rs1789693, rs34550074	iSNP, iSNP, cSNP	Abiraterone		^(72)^
		rs12422149	cSNP	Abiraterone		^(73)^
*YB-1*	Androgen receptor regulator	rs10493112	iSNP	Abiraterone		^(74)^
*CYB5A*	CYP17A1 activity regulator	rs1790834	iSNP	Abiraterone		^(75)^
*TSPYL1*	CYP17A1 and CYP3A4 regulator	rs3828743	cSNP	Abiraterone		^(76)^
*SULT1E1*	Estrogen metabolism	Group 1 (rs3775777, rs4149534, rs10019305)	iSNP	Abiraterone		^(77)^
		Group 2 (rs3775770, rs4149527, rs3775768)				

SNP, single-nucleotide polymorphism

Interestingly, several genes are overlapping in association with the prognosis between primary ADT and ARPIs, which both target the AR pathway (Table 4). SNPs in *CYP17A1* and *YB-1* are associated with the outcome of primary ADT and ARPIs, although the SNPs in each gene are different. Furthermore, the cSNP (rs1047303) in *HSD3B1*, cSNP (rs523349) in *SRD5A2*, and SNPs (rs1077858, rs1789693, and rs12422149) in *SLCO2B1* were shown to be common prognosticators in both primary ADT for HSPC and ARPIs for CRPC. The prognostic and antitumor impacts of the cSNP (rs523349) in *SRD5A2* and SNPs (rs1077858, rs1789693, and rs12422149) in *SLCO2B1* were consistent between primary ADT and abiraterone. Intriguingly, a variant allele in *HSD3B1* (rs1047303) was differentially associated with prognosis in patients treated with abiraterone and other therapies. These findings suggest that *HSD3B1* (rs1047303) may be a promising marker to select appropriate combination therapy with ADT. Further studies on the prognostic impact of these SNPs will be important to evaluate candidates for personalized medicine.

**Table 4.** Genetic Polymorphisms Associated with the Outcomes of Multiple Treatments.

Gene name	rs number	Treatment regimen	Risk allele	Outcome	Reference
*CYP17A1*	rs6162	ADT	G	OS	^(12)^
	rs743572	ADT	A	OS	^(13)^
		ADT	A	PFS	^(14)^
	rs2486758	Abiraterone	C	PFS	^(68)^
		Abiraterone	C	PFS	^(69)^
	rs10883783	Abiraterone	A	PFS	^(70)^
*HSD3B1*	rs1047303	ADT	C	PFS, MFS, OS	^(18)^
		ADT	C	PFS	^(19)^
		ADT	C	MFS	^(20)^
		ADT	C	PFS	^(21)^
		ADT	C	PFS, OS	^(22)^
		ADT+Docetaxel	C	PFS, OS	^(22)^
		Abiraterone	Null	PFS	^(64)^
		Abiraterone	A	PFS, OS	^(21)^
		Abiraterone/Enzalutamide	C	OS	^(65)^
		Abiraterone/Enzalutamide	C	PFS	^(66)^
	rs1856888	ADT	A	PFS	^(15)^
		ADT	G	OS	^(23)^
*SRD5A2*	rs523349	ADT	G	PFS, OS	^(26)^
		Abiraterone	G	PFS	^(71)^
*SLCO1B3*	rs4149117	ADT	T	OS	^(27)^
			T	PFS	^(28)^
			T	CSS	^(29)^
		Cabazitaxel	Null	OS	^(79)^
*SLCO2B1*	rs1077858	ADT	G	PFS	^(30)^
		ADT	G	OS	^(31)^
		Abiraterone	G	MRD	^(72)^
	rs1789693	ADT	T	PFS	^(30)^
		Abiraterone	T	MRD	^(72)^
	rs34550074	Abiraterone	T	MRD	^(72)^
	rs12422149	ADT	A	CSS	^(29)^
		ADT	G	PFS	^(30)^
		ADT	G	PFS	^(32)^
		ADT	G	PFS	^(31)^
		Abiraterone	G	PFS	^(73)^
*YB-1*	rs10493112	Abiraterone	A	PFS	^(74)^
	rs12030724	ADT	A	PFS	^(39)^
		ADT	A	PFS, OS	^(40)^
*ESR1*	rs1062577	ADT	A	OS	^(12)^
	rs2234693	Docetaxel	C	PFS	^(80)^
	rs9340799	Docetaxel+Thalidomide	G	PFS	^(80)^

ADT, androgen deprivation therapy; MRD, minimal residual disease; OS, overall survival; PFS, progression-free survival

## Genetic Polymorphisms and Taxane Treatment for CRPC

The taxane docetaxel is widely used not only for prostate cancer but also for various cancers such as lung, uterine, and ovarian cancers. Many reports have demonstrated the relationship between genetic polymorphisms and the efficacy and adverse events of docetaxel therapy. Previous studies, including several in prostate cancer, reported associations between drug transport genes (*ABCB1*, *ABCC2*, *ABCG1*, *ABCG2*, *SLCO1B3*) or drug metabolism genes (*CYP1B1*, *CYP2C8*, *CYP3A4*, *CYP3A5*) with therapeutic efficacy or adverse events (Figure 2) ^(81)^. As shown in Table 5, the cSNP (rs1056836, 4326C>G, L432V) in *CYP1B1* was associated with poor response and prognosis ^(82), (83)^. In addition, SNPs in *estrogen receptor 1* (*ESR1*) were also associated with treatment efficacy in prostate cancer ^(80)^. SNPs in *ESR1* were reported to be associated with the outcome of primary ADT and taxane chemotherapy although the position of SNPs in *ESR1* is different (Table 4). Then, SNPs in *ESR1* may serve as a predictive marker for taxane chemotherapy. OATP1B3, which is encoded by *SLCO1B3*, plays a role in taxane uptake into cells and is involved in taxane resistance in prostate cancer cells. SNPs in *SLCO1B3* may be associated with the treatment efficacy of taxane ^(88)^. However, a recent study showed comparable prognosis after cabazitaxel for CRPC between genotypes in *SLCO1B3* (rs4149117) ^(79)^. Because prognostic impact in primary ADT has been shown^(27), (28), (29)^, the cSNP (rs4149117) in *SLCO3B1* may serve as a predictive marker in pharmacotherapy for prostate cancer.

**Table 5.** Genetic Polymorphisms Associated with the Outcome of Taxane Treatment for Castration-Resistant Prostate Cancer.

Gene name	Function	rs number	Polymorphism types	Treatment	Validation	Reference
*CYP1B1*	Drug metabolizing enzyme	rs1056836	cSNP	Docetaxel	Validated	^(82), (83)^
*ABCB1*	Drug excretion pump	rs1128503, rs2032582, rs1045642	cSNP	Docetaxel+Thalidomide		^(84)^
*ABCB11*	Drug excretion pump	rs7602171	iSNP	Docetaxel+Thalidomide		^(85)^
*ABCG2*	Drug excretion pump	rs2231142	cSNP	Docetaxel+Vinorelbine/Estramustine phosphate		^(86)^
*ESR1*	Steroid receptor	rs2234693, rs9340799	iSNP	Docetaxel+Thalidomide		^(80)^
*GSTP1*	Antioxidant	rs1138272	cSNP	Docetaxel+Thalidomide		^(85)^
*SLC5A6*	Transporter	rs1395	cSNP	Docetaxel+Thalidomide		^(85)^
*VEGFA*	Angiogenesis	rs1570360	rSNP	Docetaxel, Celecoxib+Cyclophosphamide		^(87)^

SNP, single-nucleotide polymorphism

## Current Research Issues and Future Prospects for Personalized Medicine

The associations between multiple SNPs and therapeutic effects of pharmacotherapy for prostate cancer have been reported, as described above. However, to date, no genetic marker has been clinically utilized in pharmacotherapy for prostate cancer, which suggests potential issues as described in the following. While some SNPs have been reproducible in validation studies, others have not yielded consistent results across studies (Table 1, 3, 5). This may be because of racial differences in the frequency of genetic polymorphisms and linkage disequilibrium (a phenomenon in which there is a correlation between genetic polymorphisms in a population). To resolve this issue, multiple studies with large populations and meta-analysis studies are required. In addition, advances in technology such as artificial intelligence may serve as a breakthrough method to resolve the complex linkage disequilibrium among individuals.

Another problem is that the data in most study cohorts were retrospectively collected in daily practice. A daily clinical follow-up generally shows deviations from the strict follow-up schedule in a clinical trial. To improve the quality of data, collecting clinical data using a strict protocol is desirable to obtain more robust findings. In addition, most studies to date have focused on target genetic polymorphisms of individual genes. Because this method may miss useful SNPs, comprehensive methods such as GWAS are required. In addition, a single marker may be not enough for accurate predictive ability, and this may be overcome by using multiple SNPs. GWASs indicated that a single SNP generally provides only a modest (odds ratio, 1.1-1.5) increased susceptibility risk of prostate cancer, where polygenic risk score (PGS) using multiple risk SNPs was developed and validated ^(89), (90)^. Therefore, the PGS approach would be useful to increase diagnostic ability.

Furthermore, the genes of SNPs associated with therapeutic outcome can be the cause of treatment resistance. Therefore, these genes are promising targets to overcome treatment resistance. Genes involved in androgen metabolism such as *CYP17A1*, *HSD3B1*, *AKR1C3*, and *SRD5A2* have been candidate targets for drug discovery and drug development, and the SNPs may be crucial in therapeutic efficacy (Table 6).

**Table 6.** Druggable Targets in Androgen Metabolism and Their Inhibitors.

Target enzyme	Inhibitor	Developmental status
CYP17	Abiraterone	Approved
	Orteronel (TAK-700)	Phase III (terminated)	
	Galeterone	Phase II (terminated)	
3β-HSD	Trilostane	Phase II (terminated)/on market for Cushing's syndrome
AKR1C3	Indometacin	Phase II/on market as NSAIDs
	N-(indolylcarbonyl)-piperidines	Phase I	
5α-reductase (types I and II)	Dutasteride	Phase II (terminated)/on market for benign prostatic hyperplasia
5α-reductase (type II)	Finasteride	On market for androgenetic alopecia

NSAID, non-steroidal anti-inflammatory drug

## Conclusion

Here, we summarized the known associations between genetic polymorphisms and the outcomes of pharmacotherapy in prostate cancer patients. Recently, multiple novel therapeutic options for HSPC have emerged, and the stratification of suitable patients for each option will be required. Genetic biomarkers such as SNPs will be beneficial for stratifying patients and for estimating the treatment́ response of an individual patient. The combination of genetic biomarkers with traditional clinicopathological parameters could improve the prognostication and the choice of the most appropriate treatment for each patient, which will be helpful in clinical decision making. Thus, personalized medicine using genetic biomarkers is expected to be realized in pharmacotherapy for prostate cancer. However, unresolved issues remain, such as inconsistent results among studies as well as the current lack of GWAS and PGS approaches, and these issues should be addressed in future research.

## Article Information

### 

This article is based on the study, which received the Medical Research Encouragement Prize of The Japan Medical Association in 2020.

### Conflicts of Interest

Masaki Shiota received honoraria from Janssen Pharmaceutical Company, Astellas Pharma, and Sanofi; Shusuke Akamatsu received grant/research support from Astellas Pharma; Shintaro Narita received honoraria from Janssen Pharmaceutical Company; Naohiro Fujimoto received honoraria from Janssen Pharmaceutical Company and Astellas Pharma and grant/research support from Astellas Pharma and Sanofi; Masatoshi Eto received honoraria from Janssen Pharmaceutical Company and grant/research support from Astellas Pharma and Sanofi.

### Sources of Funding

This work was supported by Takeda Science Foundation and Japanese Urological Association to Masaki Shiota.

### Acknowledgement

We thank Gabrielle White Wolf, PhD, from Edanz Group (https://en-author-services.edanz.com/ac) for editing a draft of this manuscript.
